# Supplementary material for: A Likelihood Approach for Real-Time Calibration of Stochastic Compartmental Epidemic Models
Source: PLoS Comput Biol. 2017 Jan 17;13(1):e1005257. doi: 10.1371/journal.pcbi.1005257 (PMC5240920; doi:10.1371/journal.pcbi.1005257)
Supplement: S1 File — (TAR.GZ) [file pcbi.1005257.s014.tar.gz › HSPH_Online-SI-Revision/EnKF07/n10-mild/n10-mild_EnKF_table8.pdf]

1	R0	{1.25046, 6.80278}	{2.44887, 8.22412}	{{0.879105, 0.71252}, {3., 17.8088}}	{111.232, 120.364}	inf	102.	104.146	{10.7767, 91.6852}	77.8094	0.396432	newly	103.	151.52	{8., 229.}	135.049	0.600253	Reff	0.913086	0.403932	{0.0567902, 0.780057}	57.3059	ar	3516.	15329.6	{14855., 15926.}	335.996
2	R0	{1.32013, 4.45847}	{2.5195, 10.8792}	{{1.70024, 1.25184}, {3., 17.8827}}	{90.8525, 202.404}	inf	44.	969.073	{826.419, 826.419}	2103.99	1.27105	newly	57.	743.46	{268., 1439.}	1205.4	0.975734	Reff	0.755774	0.474589	{0.10297, 1.0599}	60.7062	ar	4420.	17405.9	{15842., 20635.}	293.798
3	R0	{1.31785, 5.36796}	{2.53693, 5.38623}	{{1.79983, 0.504728}, {3., 13.9576}}	{92.5053, 103.708}	inf	84.	1052.36	{172.538, 1988.63}	1154.21	0.788886	newly	97.	896.25	{80., 1199.}	831.351	0.725189	Reff	0.69253	0.38439	{0.0734651, 0.517533}	58.2329	ar	4942.	17318.4	{15299., 20829.}	250.434
4	R0	{1.38801, 6.16031}	{2.43432, 7.06464}	{{0.62774, 2.59373}, {3., 11.7879}}	{98.4042, 62.2044}	inf	76.	240.313	{17.557, 178.72}	267.91	0.417829	newly	81.	226.24	{14., 201.}	248.395	0.416718	Reff	0.752718	0.395724	{0.165623, 0.634879}	56.2546	ar	4790.	14088.	{13346., 14989.}	194.112
5	R0	{1.27955, 11.57}	{2.26565, 7.59647}	{{0.161455, 3.46864}, {3., 8.42236}}	{121.931, 35.7169}	inf	144.	411.345	{18.0666, 979.237}	244.54	0.523696	newly	92.	804.17	{13., 3270.}	833.533	0.687891	Reff	0.976553	0.679911	{0.0151143, 2.9997}	90.7978	ar	3460.	17962.6	{15812., 25355.}	419.149
6	R0	{1.22184, 4.34395}	{2.59042, 6.51048}	{{2.23619, 0.297611}, {3., 16.1848}}	{120.33, 162.461}	inf	60.	657.954	{21.0044, 1524.94}	1027.93	0.701045	newly	80.	1325.6	{12., 6814.}	1606.7	0.836405	Reff	0.820343	0.499103	{0.0533351, 0.726922}	64.0869	ar	3524.	17541.4	{15451., 24032.}	397.769
7	R0	{1.23856, 4.59068}	{2.59757, 6.02415}	{{2.04433, 1.0526}, {3., 7.08753}}	{109.725, 51.2668}	inf	59.	369.216	{47.7044, 320.703}	539.795	0.776744	newly	87.	746.43	{39., 601.}	775.782	0.646126	Reff	0.795403	0.509392	{0.234178, 1.32074}	60.6061	ar	3855.	15040.	{14137., 17936.}	290.141
8	R0	{1.34531, 10.6689}	{2.39221, 12.8734}	{{0.825732, 3.34546}, {3., 19.7519}}	{99.2971, 50.009}	inf	248.	377.524	{296.657, 470.937}	63.184	0.176123	newly	164.	212.74	{85., 422.}	70.439	0.260214	Reff	0.83611	0.53992	{0.0560408, 0.9353}	39.4583	ar	4852.	16593.4	{15521., 19284.}	241.99
9	R0	{1.31775, 5.19226}	{2.266, 5.52645}	{{0.718166, 3.44425}, {3., 9.56836}}	{83.3643, 50.8749}	inf	73.	795.523	{493.169, 1144.}	991.05	0.959175	newly	85.	1101.87	{271., 2517.}	1196.32	0.886208	Reff	0.713693	0.516477	{0.117288, 1.24461}	58.2727	ar	4708.	17172.4	{15334., 21425.}	264.75
10	R0	{1.25916, 6.89207}	{2.65655, 8.44617}	{{2.07396, 1.92947}, {3., 13.8308}}	{116.19, 82.5321}	inf	69.	190.547	{19.9919, 251.753}	220.815	0.488651	newly	65.	479.77	{7., 399.}	697.308	0.677559	Reff	0.845904	0.60762	{0.132357, 1.70359}	65.9603	ar	3823.	16946.9	{15394., 21587.}	343.288
11	R0	{1.20377, 5.46566}	{2.1126, 9.54801}	{{0.132457, 2.1098}, {3., 13.9229}}	{128.741, 118.581}	inf	56.	825.192	{94.6713, 501.792}	1373.56	0.7677	newly	56.	531.34	{40., 856.}	865.714	0.669502	Reff	0.847334	0.297382	{0.0265249, 0.599204}	64.9038	ar	3114.	16161.8	{14721., 17876.}	419.005
12	R0	{1.27878, 2.55804}	{2.40522, 10.2168}	{{1.65293, 6.24189}, {3., 11.7148}}	{88.0873, 299.401}	inf	4.	570.885	{72.1903, 737.598}	14172.1	1.74062	newly	9.	332.43	{32., 500.}	3593.67	1.15279	Reff	0.778393	0.519069	{0.0640099, 1.54005}	58.9515	ar	3930.	17034.1	{15093., 23376.}	333.438
13	R0	{1.41227, 5.45696}	{2.26066, 12.8728}	{{0.750598, 9.01942}, {3., 19.7019}}	{90.5751, 142.842}	inf	53.	676.93	{267.807, 2043.86}	1182.81	0.870434	newly	51.	375.49	{71., 1274.}	643.471	0.627591	Reff	0.744831	0.510111	{0.0186014, 0.785078}	52.7803	ar	4822.	17349.2	{15402., 23869.}	259.792
14	R0	{1.32917, 7.27593}	{2.63775, 3.21401}	{{2.2128, 1.838}, {3., 3.69782}}	{100.294, 59.9278}	inf	144.	813.03	{126.462, 1228.66}	478.109	0.655112	newly	106.	1519.9	{183., 4290.}	1342.34	0.946991	Reff	0.755633	0.779672	{0.187349, 2.34759}	81.4137	ar	4758.	19440.9	{16473., 25889.}	308.594
15	R0	{1.31629, 9.97481}	{2.3863, 5.70958}	{{0.465948, 0.0194152}, {3., 14.1822}}	{112.127, 74.5083}	inf	282.	740.521	{399.822, 853.057}	167.41	0.319277	newly	192.	869.73	{183., 1699.}	358.672	0.42493	Reff	0.872437	0.340817	{0.101171, 0.463775}	62.0032	ar	4543.	17108.8	{15732., 19443.}	276.597
16	R0	{1.36735, 5.74166}	{2.35637, 5.71817}	{{0.546342, 1.09453}, {3., 7.45528}}	{91.8846, 31.6558}	inf	112.	1086.33	{325.896, 2912.39}	872.944	0.818653	newly	104.	918.4	{198., 2023.}	786.192	0.783434	Reff	0.782534	0.379071	{0.0978689, 0.589939}	51.7327	ar	4516.	17821.5	{15852., 22315.}	294.63
17	R0	{1.27524, 13.2396}	{2.47833, 8.46289}	{{1.90071, 5.02342}, {3., 11.1167}}	{101.6, 36.079}	inf	176.	1158.92	{1111.08, 1111.08}	571.762	0.844513	newly	112.	876.94	{523., 1144.}	696.411	0.873891	Reff	1.09887	0.497627	{0.0899821, 0.694505}	63.3811	ar	3731.	18859.4	{17121., 24235.}	405.477
18	R0	{1.21884, 7.71545}	{2.58619, 6.69223}	{{1.7626, 3.69164}, {3., 14.2704}}	{112.185, 56.3668}	inf	93.	378.304	{134.029, 878.383}	315.935	0.478492	newly	79.	283.56	{57., 527.}	273.418	0.553833	Reff	0.870374	0.57556	{0.0861546, 1.33369}	58.2568	ar	3080.	16259.3	{14779., 20319.}	427.9
19	R0	{1.34879, 6.71464}	{2.11005, 12.4129}	{{0.77206, 7.95071}, {3., 15.101}}	{90.4659, 84.8626}	inf	64.	264.406	{22.8384, 413.319}	341.304	0.459963	newly	49.	173.46	{9., 318.}	307.878	0.514147	Reff	0.757615	0.5812	{0.146284, 0.777595}	48.6623	ar	4585.	15474.3	{14122., 20950.}	237.499
20	R0	{1.37284, 5.75036}	{2.49205, 3.88587}	{{1.58516, 3.55805}, {3., 4.44802}}	{81.5253, 32.4238}	inf	131.	941.893	{335.991, 2147.66}	619.002	0.626001	newly	141.	1147.64	{334., 2712.}	713.929	0.693437	Reff	0.7784	0.621098	{0.0500688, 1.40278}	56.2541	ar	4750.	18845.1	{15866., 24149.}	296.739
21	R0	{1.29938, 5.36419}	{2.38266, 11.2205}	{{1.712, 0.881091}, {3., 19.7686}}	{83.3694, 177.704}	inf	52.	744.542	{752.084, 752.084}	1333.98	1.12873	newly	62.	653.32	{230., 1317.}	958.742	0.908192	Reff	0.755719	0.516089	{0.136439, 1.11915}	54.2214	ar	4337.	17418.2	{15833., 23062.}	301.619
22	R0	{1.31882, 7.47626}	{2.61711, 3.82015}	{{1.76426, 2.65814}, {3., 6.19271}}	{98.4433, 48.9029}	inf	105.	777.583	{293.202, 1816.69}	641.452	0.657364	newly	86.	1178.73	{265., 2656.}	1270.8	0.8532	Reff	0.86897	0.686347	{0.188081, 1.76409}	54.607	ar	3977.	19279.3	{16644., 26939.}	384.77
23	R0	{1.26215, 3.46375}	{2.65993, 10.3792}	{{2.14983, 6.03189}, {3., 14.3517}}	{110.746, 199.653}	inf	13.	905.036	{189.985, 1171.22}	6861.81	1.55947	newly	14.	493.49	{79., 1016.}	3424.93	1.3007	Reff	0.807776	0.499738	{0.129554, 0.893754}	66.2697	ar	3607.	17486.7	{15440., 24498.}	384.8
24	R0	{1.16712, 4.19464}	{2.87712, 2.56533}	{{2.46648, 1.00773}, {3., 5.82819}}	{146.515, 69.043}	inf	40.	104.861	{52.4296, 171.318}	162.153	0.229038	newly	56.	163.59	{55., 138.}	194.625	0.254848	Reff	0.834491	0.503455	{0.288394, 0.659374}	50.4296	ar	3000.	16191.4	{15182., 18499.}	439.714
25	R0	{1.22751, 3.34517}	{2.6298, 3.44347}	{{2.20645, 2.15737}, {3., 4.34231}}	{116.071, 29.8804}	inf	37.	434.139	{263.586, 370.271}	1077.51	0.903542	newly	58.	646.33	{227., 1741.}	1019.67	0.827273	Reff	0.861344	0.500659	{0.16641, 0.671915}	63.6406	ar	3086.	17218.4	{16044., 20540.}	457.95
26	R0	{1.18747, 5.88083}	{2.48271, 5.59883}	{{1.4284, 3.28882}, {3., 9.9851}}	{109.075, 52.2529}	inf	33.	416.041	{61.0962, 573.006}	1161.11	1.0065	newly	33.	391.79	{44., 584.}	1089.36	0.95425	Reff	0.772805	0.311057	{0.0572047, 0.481929}	68.5038	ar	3614.	15569.8	{14651., 16956.}	330.818
27	R0	{1.29023, 4.76202}	{2.61814, 6.03281}	{{2.04535, 3.2518}, {3., 9.65689}}	{102.92, 51.3863}	inf	18.	482.997	{35.1435, 306.678}	2586.44	1.07255	newly	24.	623.53	{29., 1426.}	2504.13	0.925641	Reff	0.74614	0.431156	{0.0142301, 1.48434}	80.9222	ar	4245.	16523.9	{14535., 21407.}	289.256
28	R0	{1.2258, 12.9056}	{2.89774, 8.30154}	{{2.46179, 5.35858}, {3., 8.9439}}	{136.396, 35.6749}	inf	123.	338.804	{53.9682, 451.349}	201.902	0.332234	newly	72.	454.06	{37., 741.}	551.778	0.44119	Reff	0.891402	0.917451	{0.477866, 2.46154}	54.9423	ar	3639.	13410.2	{11491., 21042.}	268.514
29	R0	{1.34702, 6.29049}	{2.40474, 7.37353}	{{0.636603, 0.0386335}, {3., 15.0005}}	{101.682, 110.249}	inf	82.	470.956	{47.0325, 610.442}	490.451	0.634868	newly	86.	747.31	{45., 2173.}	785.523	0.533235	Reff	0.773998	0.429863	{0.0197177, 0.821379}	61.8602	ar	4560.	16342.4	{15001., 18586.}	258.385
30	R0	{1.37931, 6.78741}	{2.79198, 5.87902}	{{2.35736, 4.56226}, {3., 8.8723}}	{102.419, 31.6535}	inf	58.	251.331	{19.3719, 157.133}	357.417	0.428575	newly	51.	244.38	{21., 483.}	400.314	0.492046	Reff	0.764552	0.765601	{0.321746, 1.86524}	56.2841	ar	4667.	16710.	{15080., 23786.}	258.045
31	R0	{1.16062, 4.47529}	{2.6248, 6.04858}	{{2.23519, 0.0568176}, {3., 16.6485}}	{129.117, 160.384}	inf	38.	438.193	{98.2561, 430.049}	1055.06	0.889425	newly	49.	1028.17	{35., 887.}	2008.88	0.891268	Reff	0.82288	0.635723	{0.102229, 1.81654}	59.7598	ar	3037.	17224.2	{15815., 21866.}	467.145
32	R0	{1.37647, 18.7122}	{2.77461, 4.5322}	{{2.31701, 3.27008}, {3., 7.47592}}	{101.574, 75.7795}	inf	359.	259.572	{94.5148, 261.377}	62.467	0.323453	newly	137.	617.12	{108., 1438.}	360.263	0.392372	Reff	0.886034	0.885677	{0.377515, 2.31677}	65.7464	ar	4824.	16722.7	{14492., 23139.}	246.657
33	R0	{1.35084, 6.08841}	{2.27647, 6.97879}	{{0.568973, 0.419482}, {3., 14.0914}}	{99.4378, 111.246}	inf	48.	518.789	{326.087, 634.128}	982.421	0.971416	newly	49.	966.01	{146., 2540.}	1873.04	0.938682	Reff	0.722834	0.445253	{0.110557, 0.584278}	59.3698	ar	4759.	15899.3	{14835., 17733.}	234.089
34	R0	{1.36912, 6.316}	{2.25383, 5.32338}	{{0.426222, 0.425849}, {3., 13.6393}}	{102.147, 101.286}	inf	145.	552.714	{220.111, 618.943}	284.627	0.47966	newly	140.	757.54	{100., 1818.}	452.8	0.471862	Reff	0.820103	0.276493	{0.023773, 0.464581}	73.5382	ar	4537.	15999.6	{14938., 18941.}	252.647
35	R0	{1.35831, 7.09038}	{2.4028, 6.44153}	{{0.563613, 0.259389}, {3., 9.64102}}	{102.607, 56.2744}	inf	73.	648.995	{33.1421, 1098.62}	811.987	0.641228	newly	59.	935.98	{22., 2888.}	1516.75	0.75459	Reff	0.752504	0.520534	{0.100233, 0.614482}	47.7025	ar	4667.	16947.3	{15441., 22381.}	263.131
36	R0	{1.28315, 5.89938}	{2.61939, 7.84544}	{{1.73065, 2.09}, {3., 11.6068}}	{104.137, 83.1298}	inf	40.	238.464	{76.7256, 224.829}	503.269	0.630025	newly	43.	707.54	{62., 399.}	1554.6	0.590172	Reff	0.715356	0.725993	{0.127303, 1.73021}	65.3119	ar	4460.	15909.	{14119., 21242.}	256.703
37	R0	{1.19303, 2.75113}	{2.54137, 2.86967}	{{1.74315, 2.33654}, {3., 3.34471}}	{113.018, 17.6989}	inf	5.	912.992	{65.4583, 1990.51}	18162.7	1.88671	newly	9.	1623.6	{80., 5783.}	17943.6	1.78337	Reff	0.839297	0.567808	{0.11776, 1.62623}	59.7195	ar	3142.	17747.	{15389., 25846.}	464.83
38	R0	{1.36033, 7.8088}	{2.2799, 6.51141}	{{0.489945, 0.0221293}, {3., 14.3421}}	{83.2193, 88.2437}	inf	165.	740.644	{83.4109, 1264.13}	365.049	0.440694	newly	127.	602.78	{44., 1338.}	392.567	0.596763	Reff	0.778789	0.702121	{0.0929957, 1.51974}	60.8918	ar	4941.	17286.	{15071., 22225.}	249.848
39	R0	{1.27353, 3.70845}	{2.71143, 6.45902}	{{1.85397, 1.01587}, {3., 7.5258}}	{112.906, 90.1788}	inf	7.	310.156	{77.9429, 234.87}	4332.46	1.47011	newly	14.	577.37	{85., 432.}	4026.07	1.17232	Reff	0.800796	0.719499	{0.329524, 1.53559}	59.5144	ar	3725.	15859.	{14376., 20535.}	325.744
40	R0	{1.19983, 12.1486}	{2.38018, 4.39016}	{{0.9322, 2.86656}, {3., 5.32449}}	{111.674, 63.8628}	inf	140.	510.484	{59.6919, 1425.9}	281.389	0.425532	newly	71.	525.23	{48., 1372.}	651.507	0.664354	Reff	0.902632	0.363714	{0.13229, 0.557939}	61.7431	ar	3619.	16893.6	{15541., 20154.}	366.803
41	R0	{1.20935, 2.67169}	{2.58129, 6.06162}	{{2.21049, 0.329895}, {3., 15.6301}}	{126.357, 233.146}	inf	0.1	734.411	{146.948, 643.21}	734311.	3.613	newly	0.1	1178.48	{55., 886.}	1.17838×10^{6}	3.61477	Reff	0.794664	0.568021	{0.0719595, 0.730422}	50.9813	ar	3429.	17476.8	{15602., 23135.}	409.676
42	R0	{1.23313, 4.74781}	{2.62123, 11.3027}	{{1.77663, 5.88734}, {3., 14.9466}}	{112.567, 142.28}	inf	65.	261.124	{54.9454, 265.827}	316.189	0.563892	newly	90.	186.32	{22., 254.}	147.867	0.422542	Reff	0.791546	0.649258	{0.061426, 1.17615}	53.4929	ar	3835.	16148.5	{14896., 17480.}	321.083
43	R0	{1.26105, 2.39713}	{1.86953, 5.96301}	{{0.736341, 0.793188}, {3., 10.2162}}	{89.645, 208.619}	inf	1.	549.627	{392.967, 392.967}	54862.7	2.56063	newly	1.	490.96	{189., 687.}	48996.	2.48438	Reff	0.791561	0.32462	{0.0594571, 0.595329}	62.3838	ar	3723.	16105.9	{15121., 17042.}	332.604
44	R0	{1.36948, 8.88006}	{2.50191, 12.279}	{{1.05515, 3.30715}, {3., 16.6992}}	{92.3943, 76.9256}	inf	166.	381.557	{67.2751, 585.246}	159.229	0.33932	newly	127.	291.53	{68., 345.}	175.016	0.300817	Reff	0.844011	0.475421	{0.0926066, 1.04565}	69.8581	ar	4437.	14914.7	{13707., 18341.}	236.144
45	R0	{1.34882, 3.3791}	{2.03456, 6.77882}	{{0.237522, 3.61819}, {3., 11.5198}}	{108.302, 100.61}	inf	6.	649.524	{178.969, 441.209}	10725.4	1.82348	newly	9.	988.52	{159., 610.}	10883.6	1.58311	Reff	0.755069	0.530068	{0.0102201, 0.630976}	72.1248	ar	4422.	17779.7	{16075., 19826.}	302.075
46	R0	{1.23774, 4.34917}	{2.31586, 4.74863}	{{0.986226, 1.45424}, {3., 7.55592}}	{100.472, 70.3017}	inf	30.	291.271	{73.7418, 724.608}	876.105	0.781795	newly	38.	787.89	{83., 1850.}	1976.82	0.781037	Reff	0.787698	0.570218	{0.0861598, 1.44377}	64.7383	ar	3685.	15781.5	{14430., 18522.}	328.263
47	R0	{1.34327, 4.78811}	{2.08903, 2.89005}	{{0.83109, 1.95012}, {3., 3.89869}}	{87.3435, 39.6412}	inf	76.	664.229	{141.402, 2431.21}	778.751	0.71806	newly	94.	868.08	{160., 2596.}	826.723	0.706107	Reff	0.773992	0.393352	{0.119486, 0.578286}	58.9183	ar	4517.	17465.5	{14728., 24898.}	286.66
48	R0	{1.24366, 4.25814}	{2.27927, 6.53276}	{{0.510884, 3.41843}, {3., 9.90841}}	{99.1532, 72.7439}	inf	38.	491.189	{88.0355, 436.056}	1192.6	0.880147	newly	50.	478.83	{86., 1225.}	859.06	0.731153	Reff	0.803653	0.539615	{0.0809917, 1.40954}	56.961	ar	3678.	15603.8	{14183., 17783.}	324.246
49	R0	{1.36017, 7.5415}	{2.4405, 3.54489}	{{0.84588, 3.16107}, {3., 4.09555}}	{99.0037, 52.9949}	inf	152.	983.672	{446.133, 2551.52}	548.826	0.624431	newly	140.	1129.92	{435., 3634.}	708.786	0.714533	Reff	0.945454	0.418682	{0.1261, 0.777996}	61.6943	ar	4178.	17713.7	{15907., 22429.}	323.976
50	R0	{1.25333, 6.63578}	{2.4044, 4.26751}	{{1.0147, 2.75189}, {3., 3.27111}}	{103.266, 62.2424}	inf	49.	1486.31	{98.4429, 3978.3}	2937.82	1.20948	newly	43.	1971.51	{87., 5983.}	4486.07	1.31519	Reff	0.846499	0.51803	{0.152816, 0.678191}	44.2696	ar	3401.	17092.2	{13981., 22760.}	402.563
